# Magnetic foreign body ingestion in pediatric patients: report of three cases

**DOI:** 10.1186/s12893-017-0269-z

**Published:** 2017-06-24

**Authors:** Jinbeom Cho, Kiyoung Sung, Dosang Lee

**Affiliations:** 1 Department of Surgery, The Catholic University of Korea, College of Medicine, 222, Banpo-daero, Seocho-gu, Seoul, 06591 Republic of Korea; 2 Department of Surgery, Uijeongbu St. Mary’s Hospital, The Catholic University of Korea, College of Medicine, 271, Cheonbo-ro, Uijeongbu-si, Gyeonggi-do Uijeongbu-si, 11765 Republic of Korea

**Keywords:** Foreign body ingestion, Magnetic foreign body, Pediatric, Case report

## Abstract

**Background:**

Although foreign bodies (FBs) typically pass spontaneously and uneventfully through the digestive tract, a subset of such bodies may become trapped, eventually leading to significant injury. In particular, the ingestion of magnetic materials can cause serious morbidity due to proximate attraction through the intestinal wall.

**Case presentation:**

We recently treated three pediatric patients who had ingested several magnetic foreign materials. None of these patients exhibited any clinical symptoms or signs suggestive of surgical abdomen. Moreover, it was difficult to determine a definite diagnosis and a treatment plan due to limitations in history taking and radiologic examination. After admission to the hospital, these patients underwent surgery for the following reasons: (1) failure to spontaneously pass ingested foreign materials; (2) sudden-onset abdominal pain and vomiting during hospitalization; and (3) gastric perforation incidentally discovered during gastroduodenoscopy. Subsequently, all patients were discharged without complications; however, their conditions might have been fatal without surgery at an appropriate time.

**Conclusions:**

As the clear identification about the number and characteristics of ingested magnets via radiographic examination or patient history appears to be difficult in pediatric patients, close inpatient observation would be required in any case of undetermined metallic FB ingestion. Patients who are confirmed to have ingested multiple magnets should be regarded as conditional surgical patients, although their clinical conditions are stable.

## Background

Ingestion of a foreign body (FB) occurs commonly in children aged between 6 months and 6 years [1]. In the Unites States, a total of 94,820 cases were reported in 2015, and 68,371 of these cases occurred in pediatric patients aged ≤5 years [2]. Although conservative treatment is sufficient in most cases of FB ingestion [3], ingested FBs can cause serious morbidity or mortality depending on their size, their shape, and the patient’s medical status; therefore, endoscopic or surgical intervention is occasionally required. In cases involving magnet ingestion, however, clinicians often face challenges in diagnosis and management if doubt exists regarding how many magnets were ingested and whether an ingested FB is magnetic or metallic, given that clear differentiation between these two types of FBs is not always possible.

We recently treated three pediatric patients who ingested several magnetic FBs and exhibited different clinical presentations. Definitive treatment was delayed in all patients due to diagnostic uncertainty. Here, we report on these critical cases to discuss an optimized treatment strategy for magnetic FB ingestion.

## Case presentation

A comparison of the three cases is presented in Table 1.

**Table 1:** Comparison of three patients

	Patient 1	Patient 2	Patient 3
Age/ Gender	5 year/ female	28 month/ male	14 month/ male
Chief complaints at the time of admission	FB ingestion	Irritability	Irritability Vomiting
Physical examination at the time of admission	No signs of surgical abdomen	No signs of surgical abdomen	No signs of surgical abdomen
Laboratory examination at the time of admission	Normal	Normal	Normal
Initial diagnosis	Magnetic FB (the number was uncertain)	Metallic FB	Metallic FB
Initial treatment plan	Admission & observation	Admission & observation	Discharge
Time to operation	7 days	4 days	2 days
Cause to perform an operation	FB did not pass through digestive tract	Sudden-onset surgical abdomen	Incidentally discovered gastric perforation on gastroduodenoscopy
Operation findings	5 magnetic FBs Ileo-ileal fistula Cecal perforation	2 magnetic FBs Ileo-cecal fistula Internal herniation with strangulation of the ileum	3 magnetic FBs Gastro-jejunal fistula Ileal perforation
Operation	Primary closure	Primary closure Ileum resection	Primary closure
Outcome	Discharged on 7th POD	Discharged on 10th POD	Discharged on 8th POD

*FB* foreign body, *POD* post-operative day

### Patient 1

**Fig. 1:**
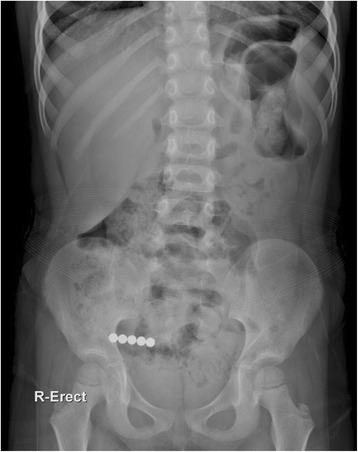
Abdomen radiography showed beaded foreign material on right lower quadrant

**Fig. 2:**
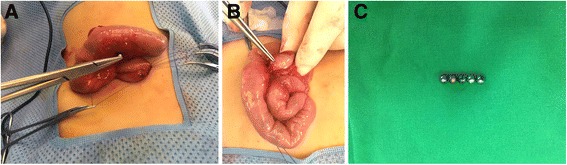
**a**, **b**, **c** Operative findings

A 5-year-old girl was admitted to the emergency medical center of our hospital with the complaint of magnetic FB ingestion. Her mother reported that she had ingested several small magnetic marbles; however, no further information regarding the exact number and size of these marbles was available at that time. It was also uncertain whether the patient had ingested magnetic FBs separately or at one time. Abdominal radiography revealed beaded FBs in the right lower quadrant (Fig. 1), suggesting five spherical bodies that had adhered to each other. Since the patient’s vital signs were stable and there were no signs of peritonitis, we opted not to perform surgical or endoscopic intervention. Instead, the patient was closely monitored for the spontaneous passage of FBs and any clinical deterioration. On the second day of hospitalization, the patient’s clinical condition, including vital signs, physical signs, symptoms, and laboratory results, remained stable; therefore, she resumed oral intake. During her hospitalization, we observed well-tolerated oral feeding with adequate defecation and good physical activity. However, serial abdominal radiographs obtained through the sixth hospital day indicated that the patient’s FBs had not passed through the digestive tract. Therefore, on the seventh day after admission, we performed diagnostic laparotomy and found multiple ileo-ileal fistulas caused by the FBs’ magnetic forces and one perforating lesion of the cecum. However, peritoneal contamination was absent due to massive adhesion of the cecum, terminal ileum, and ileum (Fig. 2a and b). Since the margin of perforation was clear and there was no evidence of intestinal necrosis, primary closure was successfully performed, and five magnetic FBs (Fig. 2c) were completely removed from the peritoneal cavity. The patient was discharged on the seventh post-operative day without any complications.

### Patient 2

**Fig. 3:**
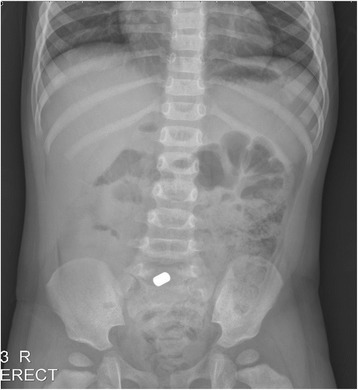
Abdomen radiography showed metallic foreign body in the abdominal cavity

**Fig. 4:**
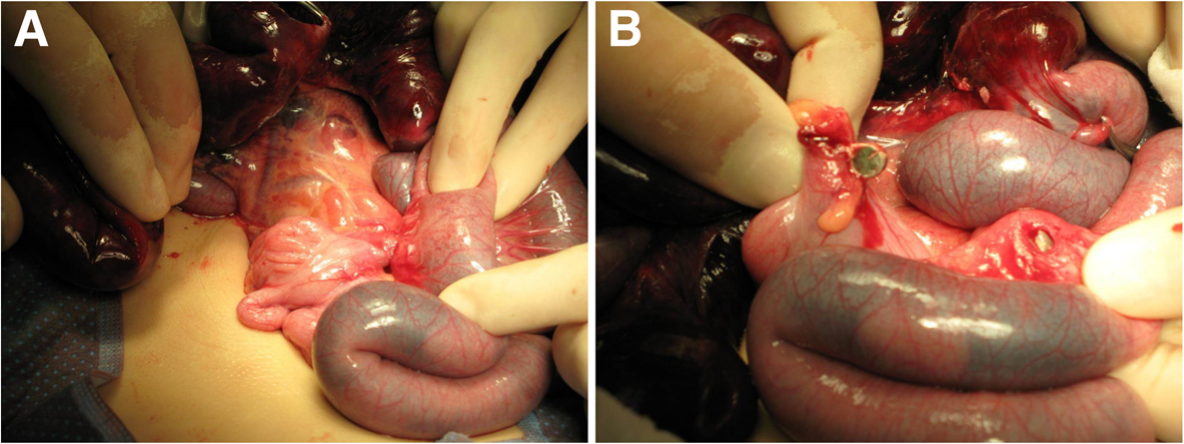
**a**, **b** Operative findings

**Fig. 5:**
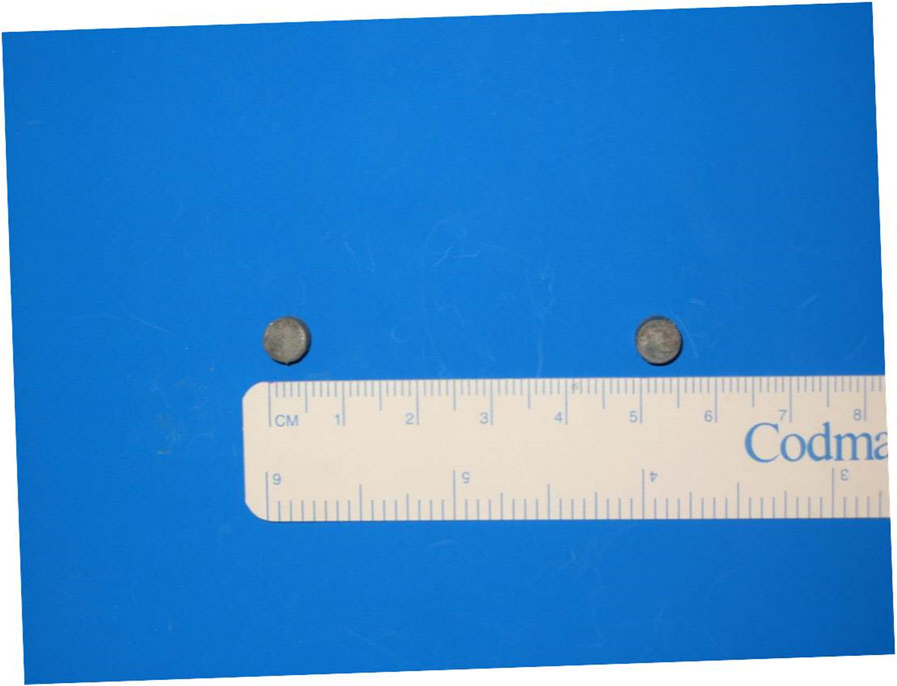
Retrieved magnetic foreign materials

A 28-month-old boy was admitted to our emergency medical center based on a complaint of irritability. At admission, his vital signs were stable, and there were no indications of abdominal tenderness or rebound tenderness suggestive of peritonitis. Abdominal radiography revealed a metallic FB in the abdominal cavity (Fig. 3). However, no information regarding this FB was available at that time. Because the boy was stable and no signs of surgical abdomen were observed, we planned to perform conservative treatment with close monitoring until the FB was spontaneously passed. However, on the fourth day of hospitalization, the patient exhibited abdominal distention and projectile vomiting with markedly reduced physical activity. Therefore, emergent surgery was performed under the suspicion of a surgical emergency. The abdominal cavity was entered via a mid-line incision, and we found an internal herniation of the small intestine through a space created by adhesion of the cecum and the ileum (Fig. 4a and b). The metallic FB that had been identified on the abdominal radiograph taken on the day of admission was demonstrated to be two magnetic FBs (Fig. 5) that were separately located in the cecum and the terminal ileum. The attachment of these two FBs caused an ileocecal fistula and eventually led to internal herniation of the small intestine. Consequently, we resected the necrotic small intestine and performed end-to-end anastomosis and primary repair for the perforating lesions of the terminal ileum and the cecum. The patient was discharged on the 10th post-operative day without complications.

### Patient 3

**Fig. 6:**
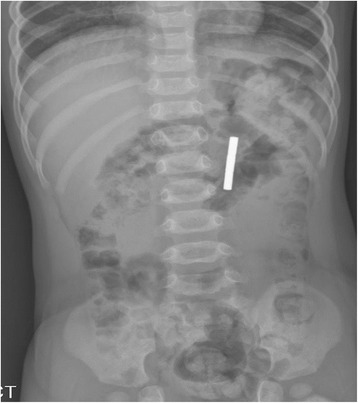
Abdomen radiography showed rod shaped metallic foreign body on left upper quadrant

**Fig. 7:**
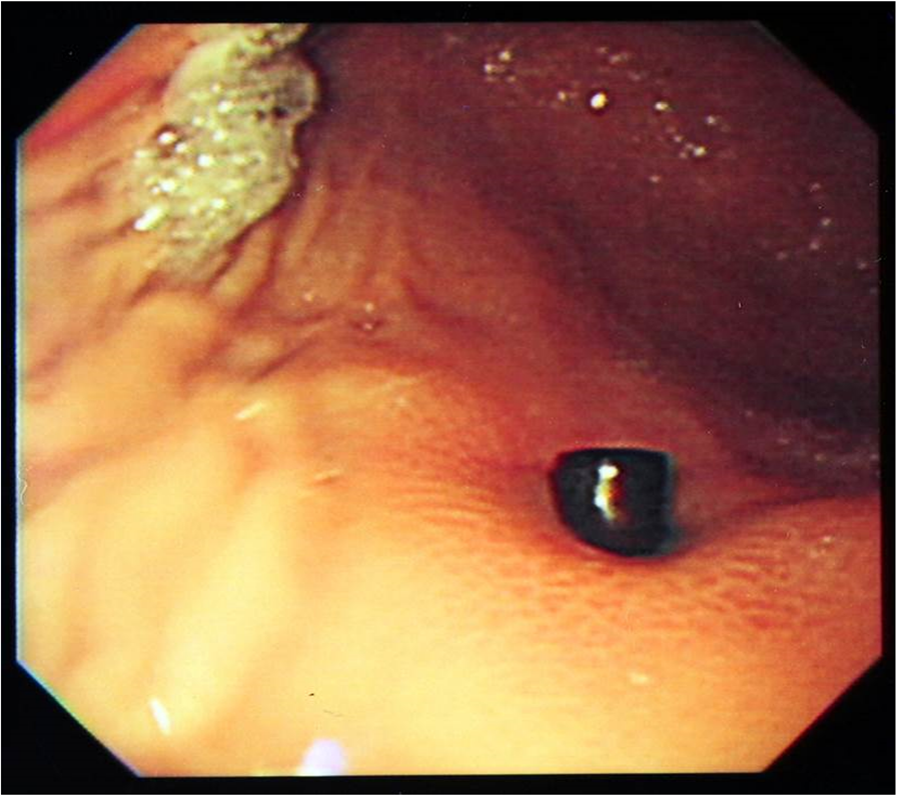
Gastric perforation discovered on diagnostic gastroduodenoscopy

**Fig. 8:**
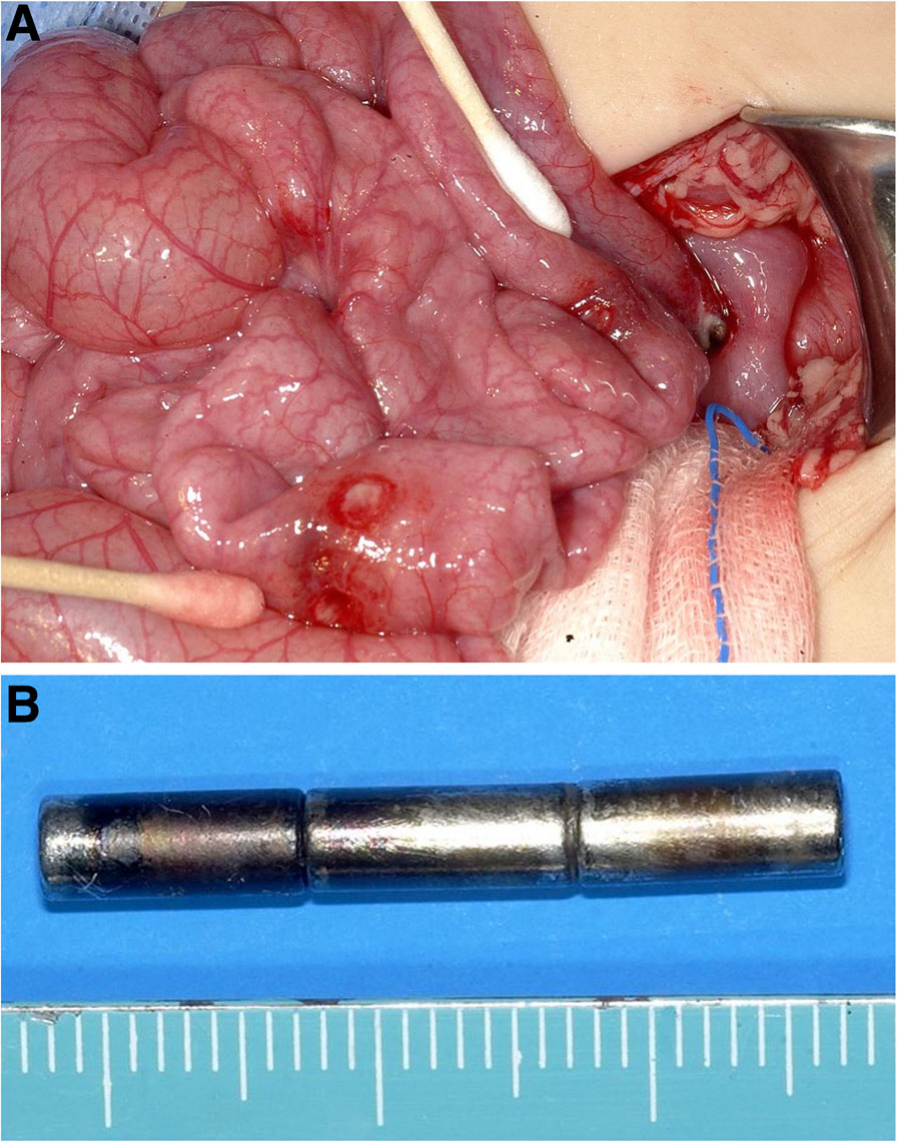
**a**, **b** Operative findings

A 14-month-old boy was admitted to our emergency medical center with the complaint of vomiting and irritability. Abdominal radiography revealed a metallic FB in the abdominal cavity (Fig. 6). However, no information regarding this FB was available at that time. Since the patient’s clinical condition became stable after fluid infusion with short-term bowel rest and he exhibited good physical activity with no abdominal pain, we discharged him from the emergency medical center after educating his parents to carefully look for spontaneous passage of the FB. However, the patient revisited our hospital for persistent postprandial vomiting 2 days after his discharge. On abdominal radiography, the FB remained in the location indicated by the patient’s previous examination. Because no signs of surgical abdomen were observed, we decided to perform a diagnostic endoscopy to investigate whether the FB had caused gastric obstruction. However, flexible endoscopy revealed that the FB had perforated the gastric wall (Fig. 7). Therefore, the boy was immediately transferred to the operating room, and emergent surgery was performed. The abdominal cavity was entered via a mid-line incision, and we found that the metallic FB observed via radiography was actually three attached magnetic FBs; one of these FBs was located in the body of the stomach, and the other two FBs were in the jejunum. The adherence of these three FBs caused a gastrojejunal fistula with adhesion and perforation of the ileum (Fig. 8a and b). Because the patient’s perforating lesions were small and had clear margins, we successfully performed primary repair of the stomach, jejunum, and ileum. The patient was discharged on the eighth post-operative day without complications.

## Discussion

In pediatric patients, ingested FBs are typically small objects such as coins, fish bones, marbles, and drugs; a recent meta-analysis indicated that batteries and sharp objects should be removed immediately but that other ingested FBs can be passed spontaneously [4]. In particular, the accidental ingestion of magnetic FBs in pediatric patients has become common due to the increasing use of toys with magnetic elements [5]. A single magnetic FB does not cause serious morbidity because it simply behaves as an isolated FB; in contrast, multiple magnetic FBs can attract each other across intestinal walls, leading to intestinal obstruction, fistulas, or perforation. Prior case reports have recommended the endoscopic or surgical removal of ingested magnets by clinicians before FB-associated symptoms develop [6, 7]. Literature reviews have revealed that prompt surgical intervention is required in cases involving the ingestion of multiple magnetic FBs and any symptoms indicative of surgical abdomen but that conservative treatment might be appropriate in cases involving the ingestion of a single magnetic FB and no definite evidence of intestinal obstruction or perforation [8, 9]. However, we found a lack of established consensus regarding treatment strategy in certain situations; in particular, the following questions remain unresolved. (1) What treatment can we perform if patients have ingested multiple magnetic FBs but exhibit no specific clinical symptoms? (2) How long can we safely wait for the spontaneous passage of ingested FBs in patients who have ingested one or more magnets? (3) What should we do if there is uncertainty regarding the number or characteristics of ingested FBs?

As presented in our case reports, it appears to be difficult to determine a treatment plan for pediatric patients with FB ingestion because children and babies often cannot explain where they have pain or how their pain feels. Moreover, they cannot describe the number, size, or nature of ingested FBs. Therefore, clinicians usually tend to depend on parental reports. However, clear history taking is not always possible. We found that patient or caregiver reports appear to be less important in pediatric patients than in adult patients. Instead, radiologic examinations might play a key role in establishing an initial diagnosis and treatment strategy for pediatric patients. However, as evidenced by our second and third cases, abdominal radiography cannot differentiate between attached multiple magnets and a single metallic FB unless images of an FB reveal a distinctive feature (i.e., a beaded appearance). Moreover, it seems to be impossible to detect whether the magnets are in physical contact via plain radiographs [10]. Consequently, clinicians appear to have no actual means of determining a definite diagnosis in pediatric patients with FB ingestion unless patients’ caregivers provide correct and definite information. Additionally, stable clinical condition does not guarantee stable intra-abdominal condition because intestinal contents or inflammatory exudate might be contained in a localized peritoneal space due to massive intestinal adhesion; accordingly, signs of peritoneal irritation signs might be absent, as observed in the first case. In this case, the patient might be fatal if we had discharged her or if empirical surgical intervention had not been performed. Therefore, we cautiously provide the following recommendations for the treatment of pediatric patients with FB ingestion: (1) if an ingested FB is metallic on radiologic examination or if the number and nature of ingested FBs cannot be determined, a surgical specialist should be consulted to reach a decision regarding endoscopic or surgical intervention; (2) if a patient exhibits signs of surgical abdomen, including abdominal distension, pain, and vomiting, prompt removal of ingested FBs regardless of their number or nature should be considered; and (3) patients who are confirmed to have ingested multiple magnets should be regarded as conditional surgical patients and should be observed closely for any clinical deterioration even if they appear to be clinically stable. However, it remains challenging to determine the appropriate time to perform a diagnostic operation for patients who fail to evacuate their ingested FBs after hospitalization and oral feeding. We suggest that it is best to remove ingested magnets before FB-associated symptoms develop; therefore, medical teams must adequately discuss treatment options with patients and their families.

## Conclusions

In conclusion, close inpatient observation should be conducted for any pediatric patients with magnet ingestion, with the exception of patients who are confirmed to have ingested a single magnet by their caregiver or via radiologic examination. Premature discharge from the hospital due to a misdiagnosis or the misconception that a solitary magnet has been ingested may lead to a fatal outcome. Early surgical or endoscopic intervention performed before symptoms develop or at the first sign of surgical abdomen could prevent more severe complications.

## Abbreviation

FB: Foreign body
